# Exploring the influencing factors of non-insulin drug prescriptions in discharged patients with type 1 diabetes

**DOI:** 10.3389/fendo.2024.1381248

**Published:** 2024-09-25

**Authors:** Yikang Cheng, Haizhen Li, Xin Liu, Xiaolong Jin, Junming Han, Jing Du, Chao Xu

**Affiliations:** ^1^ The First Clinical Institute, Zunyi Medical University, Zunyi, China; ^2^ Department of Endocrinology, Shandong Provincial Hospital Affiliated to Shandong First Medical University, Jinan, China; ^3^ Department of Endocrinology, Dongying City District People Hospital, Dongying, China; ^4^ Department of Endocrinology and Metabolism, Dongying People's Hospital, Dongying, China; ^5^ Department of Endocrinology, The First Affiliated Hospital of Baotou Medical College, Inner Mongolia University of Science and Technology, Baotou, China; ^6^ Department of Endocrinology, Shandong Provincial Hospital, Shandong University, Jinan, China

**Keywords:** type 1 diabetes, factor, insulin, prescriptions, therapy

## Abstract

**Objective:**

The aim of this study was to evaluate the admission indicators and characteristics of individuals diagnosed with type 1 diabetes (T1D) to ascertain potential impact on the choice of glucose control therapy after discharge.

**Methods:**

A total of 398 eligible T1D patients were selected. We conducted multivariable logistic regression analysis to determine the independent influence of predictors on the selection of glucose control therapy after discharge. To explore the influencing factors of different subgroups, we additionally performed subgroup analyses based on gender and age.

**Results:**

Our study revealed that body mass index (BMI) was noteworthy influence factor for prescription of insulin and non-insulin antidiabetic drug (NIAD prescription) in T1D patients of general population [OR = 1.109 (1.033-1.195), *p* = 0.006], male [OR = 1.166 (1.040−1.318), *p* = 0.011] and individuals below the age of 30 years [OR = 1.146 (1.020−1.301), *p* = 0.028]. Diastolic blood pressure (DBP) was a protective factor for NIAD prescription in the general population [OR = 0.971 (0.949-0.992), *p* = 0.008] and women [OR = 0.955 (0.923−0.988), *p* = 0.008]. The other risk factor of NIAD prescription in men was dyslipidemia [OR = 4.824 (1.442−22.246), *p* = 0.020]. Pulse pressure [OR = 1.036 (1.007–1.068), *p* = 0.016] constituted an additional risk factor of NIAD prescription among individuals below the age of 30 years. The risk factors of NIAD prescription for people aged 30 to 50 years were length of stay [OR = 1.097 (1.014–1.196), *p* = 0.026] and initial blood glucose [OR = 1.078 (1.007–1.168), *p* = 0.047]. In the case of individuals aged above 50 years, physicians exhibited a higher tendency to prescribe supplementary non-insulin medications to men [OR = 9.385 (1.501–87.789), *p* = 0.029].

**Conclusions:**

We identified notable factors that influence discharge prescriptions in patients with T1D. In order to enhance the treatment outcome for the patient, clinicians ought to have a special focus on these indicators or factors.

## 1 Introduction

Type 1 diabetes (T1D) is an insulin-dependent condition caused by the destruction of pancreatic β-cells, which causes insufficient insulin secretion in the body (1). Approximately 5% to 10% of diabetes cases worldwide are type 1, but its incidence has increased over the past few decades (2, 3). At present, exogenous insulin substitution therapy has become the main treatment for T1D. The systemic adverse effects associated with insulin therapy primarily encompass hypoglycemia, weight gain, edema, refractive error, and anaphylaxis (4).

Hypoglycemia makes it less easy to achieve good results through intensive therapy (5). In addition, intensive therapy can also lead to an increased prevalence of obesity in individuals with T1D (6). Chronic pro-inflammation induced by obesity contributes to the development of insulin resistance (7). A considerable number of patients continue to fall short of attaining optimal glycemic control (8). Several studies have demonstrated the efficacy of metformin in the maintenance or reduction of weight in patients with T1D, as well as its potential to decrease insulin dosages (9–11). GLP-1 receptor agonists (12–14) and sodium-glucose cotransporter 2 inhibitors (15–18) have exhibited effectiveness in facilitating weight loss and diminishing insulin dosage, while also enhancing glycated hemoglobin levels (HbA1c), albeit concurrently elevating the likelihood of ketosis. Non-insulin drugs are not typically recommended in clinical practice, and treatment should be based on insulin therapy and individual patient needs, but what makes a clinician decide to use non-insulin medication? We do not know yet.

People diagnosed with T1D usually require hospital admission due to the occurrence of a sudden onset or certain acute complication (4). Do certain indicators or characteristics observed at admission impact the selection of the discharge treatment plan? The prognoses of T1D have long been different between different genders and ages (19–21). Do these indicators or characteristics have different effects on the choice for different gender and age groups? Hence, a study was undertaken with the primary objective of assessing the admission indicators or characteristics of individuals diagnosed with T1D, aiming to determine their potential influence on the therapy of glucose control upon discharge. As a result, healthcare practitioners are encouraged to prioritize these indicators or characteristics to optimize treatment plans and improve patient outcomes during therapy.

## 2 Materials and methods

### 2.1 Study subjects

We first selected 445 T1D patients hospitalized in Shandong Provincial Hospital affiliated to Shandong First Medical University (Jinan, China) from January 2008 to December 2018. In the case of multiple hospital records, it is customary to retain only one. Patients with too many missing indicators or features would also be excluded from the study. Finally, this retrospective study comprised a total of 398 patients selected based on exclusion criteria. We conducted a comparative analysis between the group treated with insulin and non-insulin antidiabetic drug (NIAD group) and the group treated with insulin alone (INS group).

This retrospective study used anonymized data with no need for obtaining informed consent from each patient. The Ethics Committee of Shandong Provincial Hospital affiliated to Shandong First Medical University approved the project.

### 2.2 Measurements

The researchers obtained all data from the archival hospital records of patients admitted to Shandong Provincial Hospital affiliated to Shandong First Medical University. Upon admission, healthcare professionals perform a comprehensive assessment, including a thorough medical history review, physical examination, and analysis of blood samples. The calculation of BMI (kg/m^2^) involves dividing weight by the square of height. Blood pressure was measured following a 5-min period of rest using an electronic sphygmomanometer (HEM-7117; Omron, Kyoto, Japan). The total cholesterol (TC), triglycerides (TG), low-density lipoprotein (LDL), high-density lipoprotein (HDL), glutamyl transferase (GGT), aspartate aminotransferase (AST), glutamic pyruvic transaminase (ALT), blood glucose, albumin, potassium (K), sodium (Na), chlorine (CI), calcium (Ca), and phosphorus (P) were analyzed using an automatic biochemistry analyzer (Beckman Coulter Analyzer AU58 Series, USA). Glycated hemoglobin (HbA1c) levels were measured using high-performance liquid chromatography (HPLC) with a hemoglobin A1c analyzer manufactured by TOSOH Corporation, Japan. Hemoglobin (Hb) levels were analyzed using a blood cell analyzer (Sysmex Corporation XN-2100, Japan).

### 2.3 Statistical analysis

Initially, descriptive statistical methods were employed to provide a comprehensive summary of the clinical characteristics and biological indicators observed in the patients. The Kolmogorov–Smirnov test is used to assess the normality of continuous variables prior to conducting parameter tests. Continuous parameters that follow normal distribution are presented as the mean ± standard deviation (SD). Continuous parameters that do not conform to a normal distribution are presented as the median within the interquartile range. Categorical variables are presented in the form of percentages. The entire sample was partitioned into the NIAD group and INS group in order to conduct statistical analysis and construct a model. In order to display the distinction between the NIAD group and the INS group, the Mann–Whitney *U*-test was employed for continuous variables that do not conform to a normal distribution. The chi-square test or Fisher’s exact test was used to examine the categorical variables.

We conducted multivariable logistic regression analysis to ascertain the independent impact of predictors on the selection of blood glucose control program upon discharge among patients with T1D. The predictors of interest, including age, sex, BMI, smoking history, drinking history, family history of diabetes, dyslipidemia, osteoporosis, SBP, DBP, fatty liver, length of stay and initial blood glucose, are sequentially incorporated into the multivariate logistic regression model. To investigate potential variations in influencing factors across distinct genders and age groups, we additionally developed stepwise logistic regression models for each gender and age group. A two-sided *p*-value < 0.05 was considered statistically significant. R software (version 4.2.2) was used for all statistical analyses.

## 3 Results

**Figure 1:**
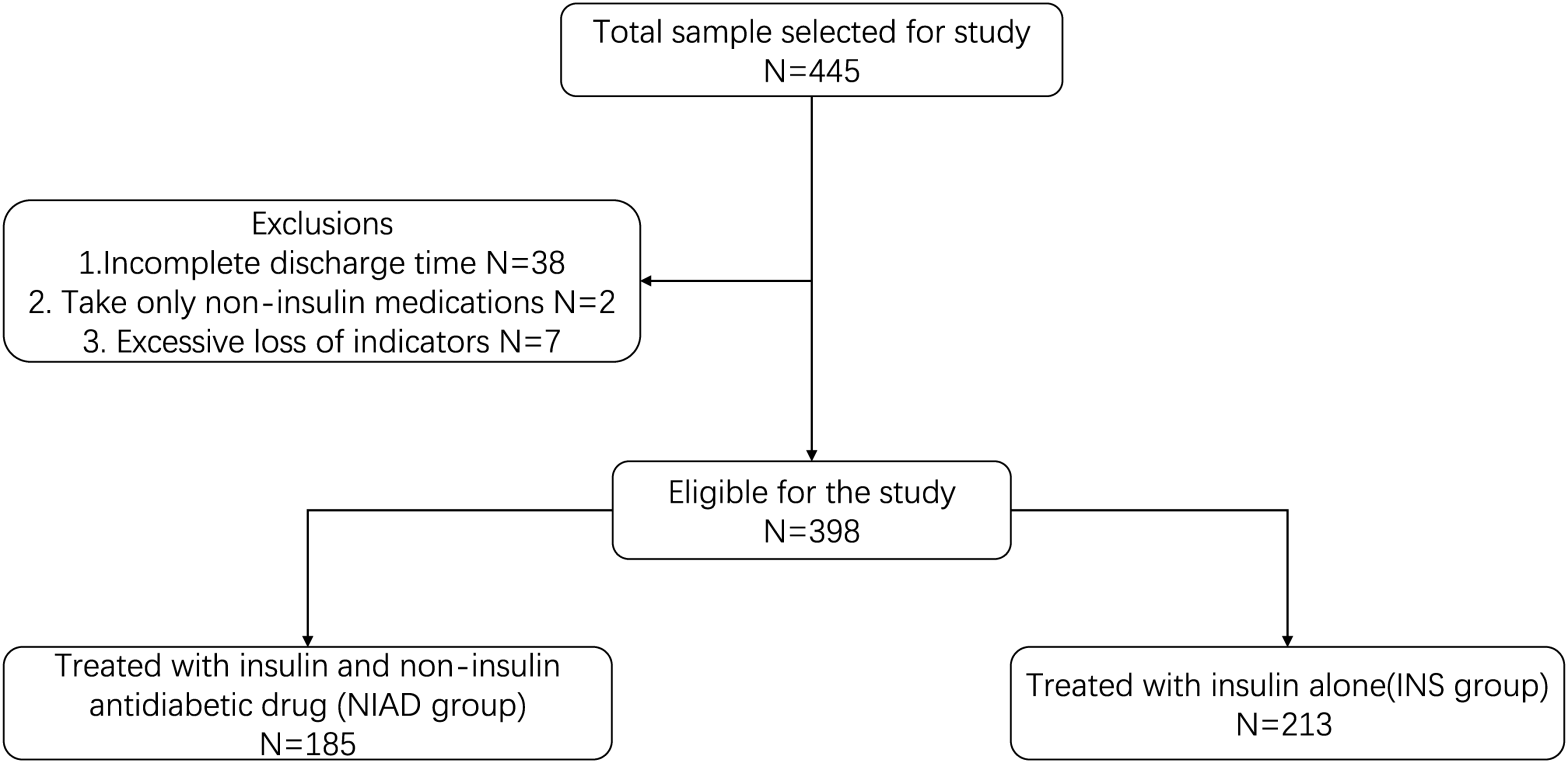
Flowchart of the study population.

This study enrolled 398 (NIAD: 185; INS: 213) patients with T1D (**Figure 1**). The median age of the study population was 30 (21–44). Male patients accounted for approximately 45.72% of the study population. The median length of stay (LOS) was 11 (8–14).

After analysis of the baseline characteristics of the patients, variables with statistical differences between the NIAD and INS groups included sex (*p* < 0.01), age (*p* < 0.01), diabetic peripheral vascular disease (*p* < 0.01), diabetic retinopathy (*p* = 0.02), fatty liver (*p* = 0.03), drinking history (p < 0.01), initial blood glucose (*p* = 0.01), BMI (*p* < 0.01), Hb (*p* = 0.03), GGT (*p* = 0.02) , LOS (*p* < 0.01) (**Table 1**).

**Table 1:** Baseline study population characteristics stratified by insulin and non-insulin antidiabetic drugs (NIAD group) and the group treated with insulin alone (INS group) at discharge.

Characteristics	INS(n=213)	NIAD(n=185)	p-value
Sex			<0.01
Male, n(%)	81(38.03)	101(54.59)	
Female, n(%)	132(61.97)	84(45.41)	
Age(years), median (IQR)	26.00(19.00-38.00)	34.00(25.00-47.00)	<0.01
Course of disease (months), median (IQR)	36.00(4.00-108.00)	36.00(5.00-108.00)	0.82
Hyperglycemia symptoms n = 392, n(%)	178(85.58)	144(78.26)	0.08
DK/DKA, n(%)	126(59.15)	93(50.27)	0.09
Infectious diseases, n(%)	55(25.82)	36(19.46)	0.17
Number of cardiovascular chronic diseases			0.41
0, n(%)	109(51.17)	82(44.32)	
1, n(%)	87(40.85)	81(43.78)	
2, n(%)	14(6.57)	19(10.27)	
3, n(%)	3(1.41)	3(1.62)	
Dyslipidemia, n(%)	21(9.86)	27(14.59)	0.20
Osteoporosis, n(%)	13(6.10)	18(9.73)	0.25
Number of chronic complications			0.14
0, n(%)	128(60.09)	88(47.57)	
1, n(%)	43(20.19)	49(26.49)	
2, n(%)	22(10.33)	23(12.43)	
3, n(%)	10(4.69)	15(8.11)	
4, n(%)	10(4.69)	10(5.41)	
Diabetic peripheral vascular disease, n(%)	35(16.43)	53(28.65)	<0.01
Diabetic retinopathy, n(%)	28(13.15)	42(22.70)	0.02
Diabetic nephropathy, n(%)	33(15.49)	33(17.84)	0.62
Fatty liver, n(%)	10(4.69)	20(10.81)	0.03
Thyroid disease, n(%)	27(12.68)	25(13.51)	0.92
Smoking history, n(%)	31(14.55)	40(21.62)	0.09
Drinking history, n(%)	17(7.98)	33(17.84)	<0.01
Family history of diabetes n=397, n(%)	58(27.23)	54(29.35)	0.72
Islet cell antibodies n=240, n(%)	20(17.24)	23(18.55)	0.92
IAA n=234, n(%)	38(33.92)	48(39.34)	0.47
GADA n=247, n(%)	34(28.33)	41(32.28)	0.59
Number of positive antibodies n=248			0.42
0, n(%)	63(51.64)	53(42.06)	
1, n(%)	33(27.05)	42(33.33)	
2, n(%)	18(14.75)	24(19.05)	
3, n(%)	8(6.56)	7(5.56)	
Initial blood glucose(mmol/L), median (IQR)	17.60(15.00-20.61)	16.13(14.00-19.97)	0.01
BMI(kg/m2), median (IQR)	19.60(17.80-22.27)	21.48(19.59-23.99)	<0.01
SBP(mmHg), median (IQR)	119.00(108.00-132.00)	123.00(110.00-138.00)	0.13
DBP(mmHg), median (IQR)	79.00(70.00-88.00)	77.00(69.00-84.00)	0.06
RBG(mmol/L), median (IQR)	13.30(8.43-18.57)	13.02(8.57-17.87)	0.74
HbA1c(%), median (IQR)	11.20(9.00-13.60)	11.07(9.10-12.90)	0.39
Hb(g/L), median (IQR)	133.00(122.00-146.00)	138.00(126.00-150.00)	0.03
AST(U/L), median (IQR)	19.00(15.00-26.00)	18.00(15.00-24.00)	0.27
ALT(U/L), median (IQR)	15.00(11.00-25.00)	17.00(13.00-23.00)	0.13
GGT(U/L), median (IQR)	14.00(11.00-20.00)	16.00(12.00-21.00)	0.02
Albumin(g/L), median (IQR)	39.50(34.40-42.40)	40.30(36.10-42.70)	0.06
TC(mmol/L), median (IQR)	4.64(3.92-5.68)	4.66(4.03-5.55)	0.69
HDL(mmol/L), median (IQR)	1.29(1.09-1.49)	1.30(1.09-1.55)	0.72
LDL(mmol/L), median (IQR)	2.65(2.09-3.42)	2.67(2.16-3.36)	0.89
TG(mmol/L), median (IQR)	0.97(0.69-1.39)	1.03(0.74-1.51)	0.12
K(mmol/L), median (IQR)	4.20(3.90-4.50)	4.10(3.90-4.50)	0.30
Na(mmol/L), median (IQR)	138.00(135.00-140.60)	139.00(136.00-140.90)	0.07
CI(mmol/L), median (IQR)	103.70(101.00-106.00)	104.00(101.00-106.90)	0.16
Ca(mmol/L), median (IQR)	2.35(2.21-2.45)	2.32(2.20-2.42)	0.09
P(mmol/L), median (IQR)	1.24(1.00-1.42)	1.16(0.98-1.34)	0.06
LOS(days), median (IQR)	10.00(8.00-13.00)	11.00(9.00-14.00)	<0.01

DK, diabetic ketosis; DKA, diabetic ketoacidosis; IAA, insulin autoantibody; GADA, glutamic acid decarboxylase antibody; SBP, systolic blood pressure; DBP, diastolic blood pressure; RBG, random blood glucose; HbA1c, glycosylated hemoglobin; Hb, hemoglobin; AST, aspartate aminotransferase; ALT, glutamic pyruvic transaminase; GGT, glutamyl transferase; TC, total cholesterol; HDL, high-density lipoprotein; LDL, low-density lipoprotein; TG, triglycerides; K, potassium; Na, sodium; CI, chlorine; Ca, calcium; P, phosphorus; LOS, length of stay.

**Figure 2:**
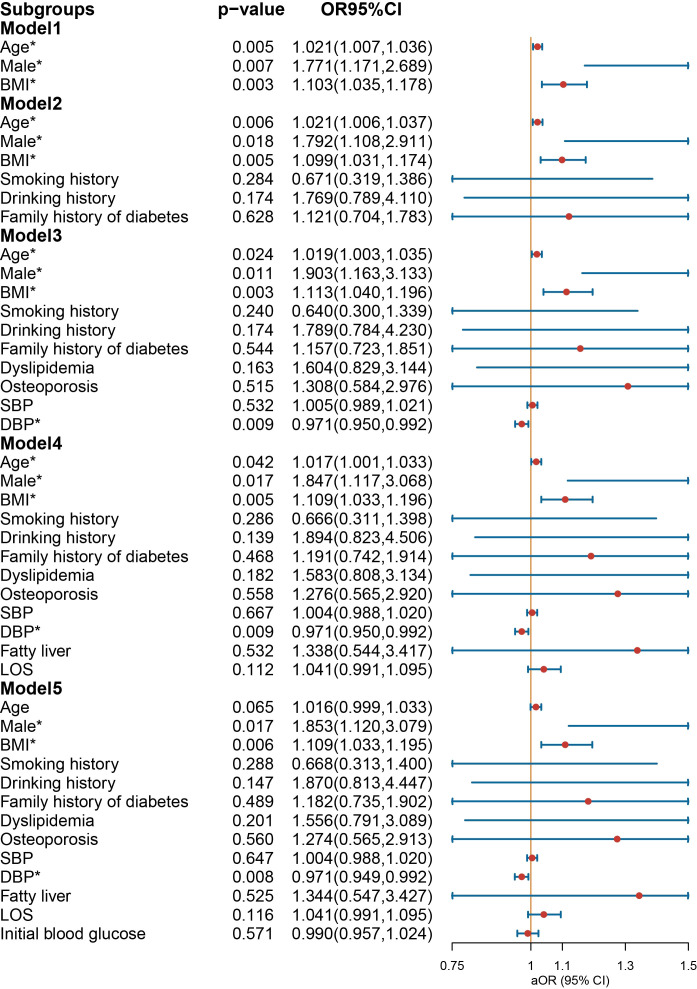
Odds ratio [95% confidence interval] of being discharged with insulin and non-insulin antidiabetic drug among patients with type 1 diabetes. SBP, systolic blood pressure; DBP, diastolic blood pressure; LOS, length of stay. *Statistical significance.

In the overall multivariate logistic regression (**Figure 2**), NIAD prescription was associated with older age, male, and higher BMI (Model 1). After further adjustment for history of smoking and drinking, family history of diabetes, dyslipidemia, osteoporosis, SBP, DBP, not being prescribed NIAD was associated with DBP (Model 3). After final adjustment for initial blood glucose, older age no longer associated with NIAD prescription. Male [OR = 1.853 (1.120-3.079), *p* = 0.017] and higher BMI [OR = 1.109 (1.033-1.195), *p* = 0.006] were still associated with NIAD prescription, and DBP was a protective factor for NIAD prescription [OR = 0.971 (0.949-0.992), *p* = 0.008].

**Figure 3:**
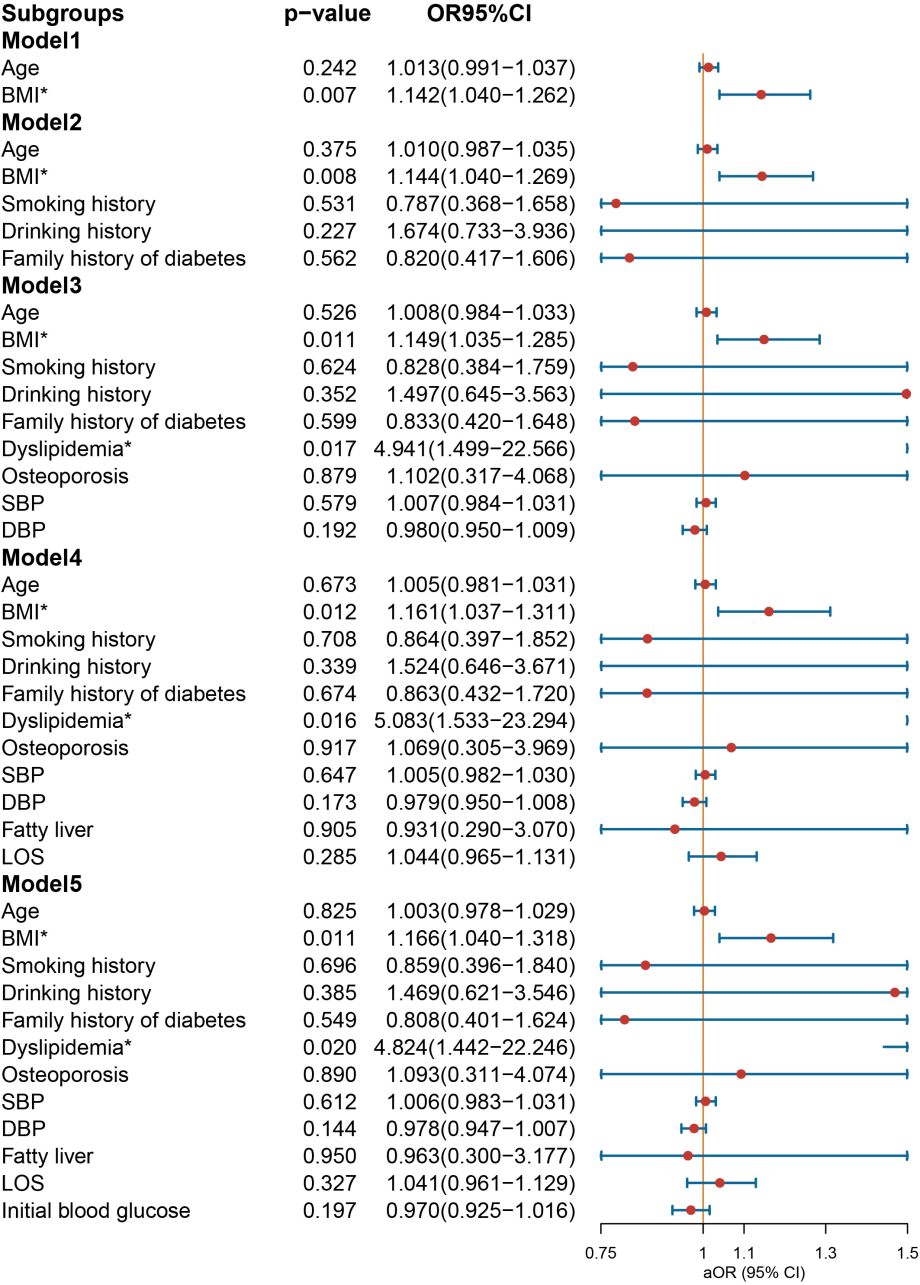
Odds ratio [95% confidence interval] of being discharged with insulin and non-insulin antidiabetic drug among male patients with type 1 diabetes. SBP, systolic blood pressure; DBP, diastolic blood pressure; LOS, length of stay. *Statistical significance.

**Figure 4:**
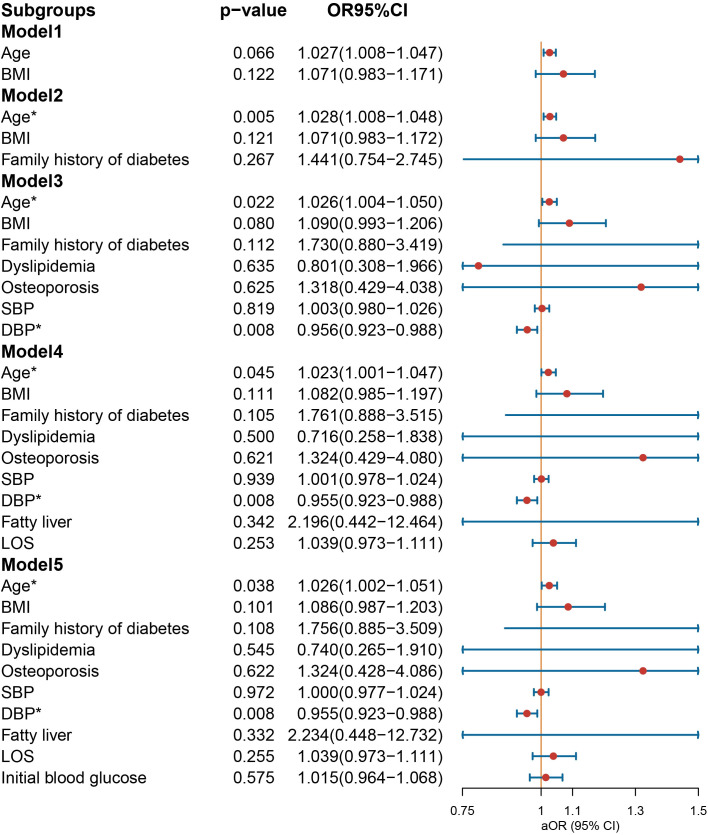
Odds ratio [95% confidence interval] of being discharged with insulin and non-insulin antidiabetic drug among female patients with type 1 diabetes. SBP, systolic blood pressure; DBP, diastolic blood pressure; LOS, length of stay. *Statistical significance.

In the multivariate logistic regression analysis conducted on the male group (**Figure 3**), NIAD prescriptions continued to exhibit a significant association with elevated BMI [OR = 1.166 (1.040−1.318), *p* = 0.011] and dyslipidemia [OR = 4.824 (1.442−22.246), *p* = 0.020] after controlling for confounding variables. In the female population (**Figure 4**), not being prescribed NIAD was associated with DBP [OR = 0.955 (0.923−0.988), *p* = 0.008]. NIAD prescriptions exhibited a significant association with older age [OR = 1.026 (1.002−1.051), p = 0.038].

**Figure 5:**
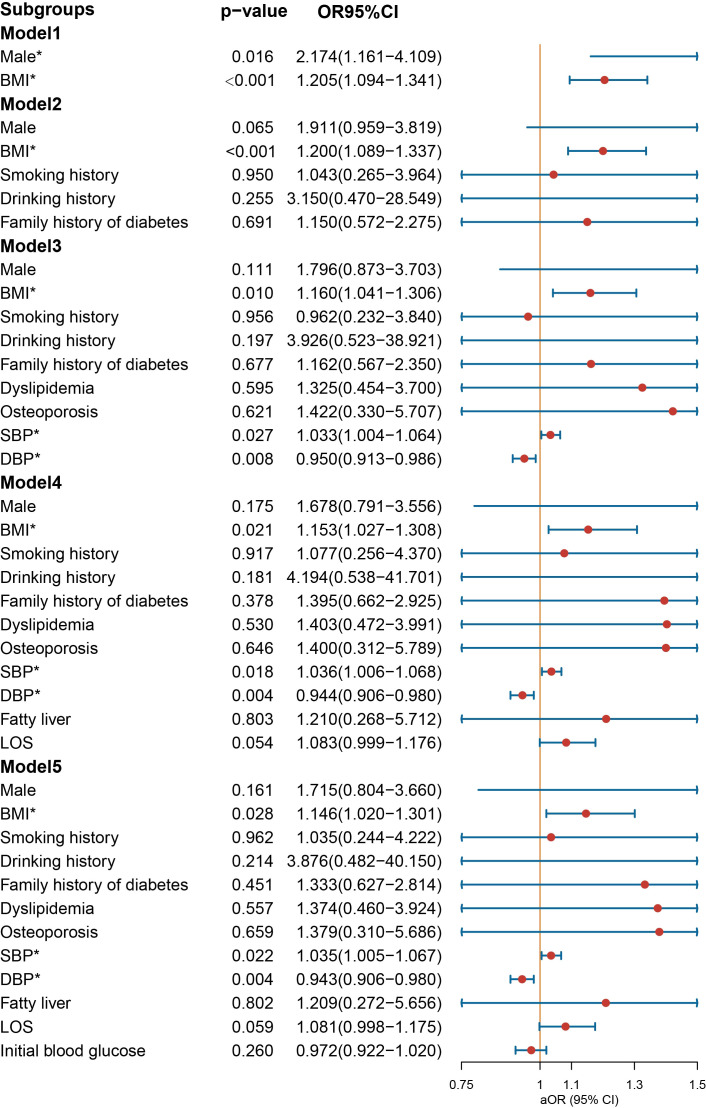
Odds ratio [95% confidence interval] of being discharged with insulin and non-insulin antidiabetic drug among patients below the age of 30 with type 1 diabetes. SBP, systolic blood pressure; DBP, diastolic blood pressure; LOS, length of stay. *Statistical significance.

**Figure 6:**
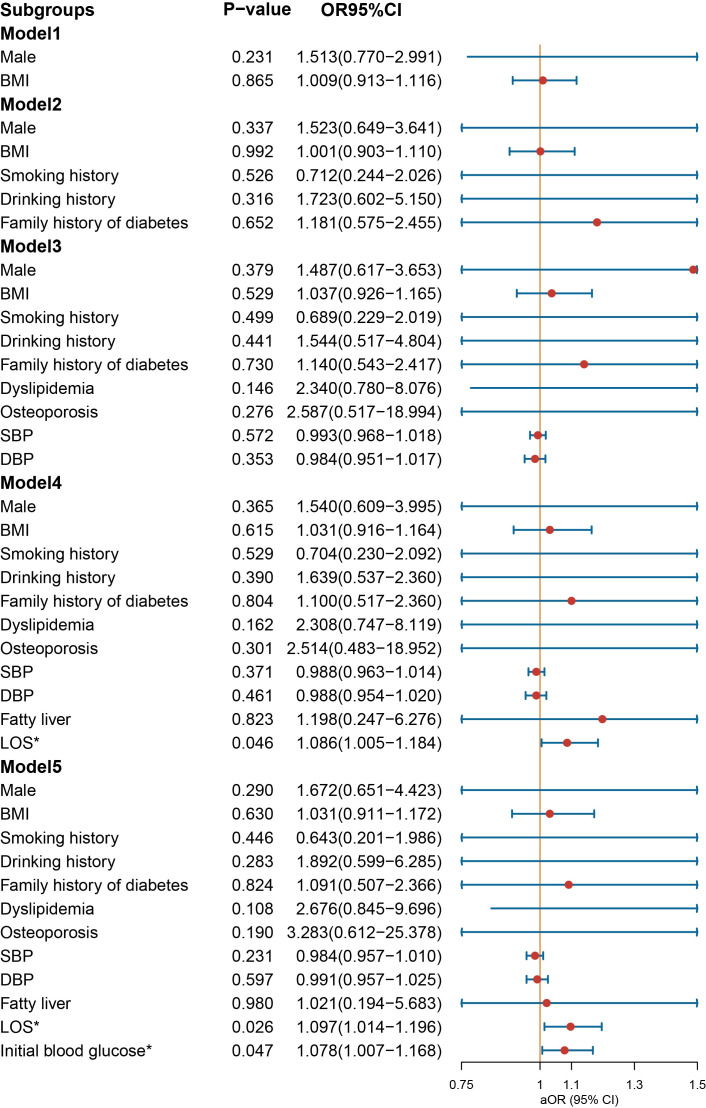
Odds ratio [95% confidence interval] of being discharged with insulin and non-insulin antidiabetic drug among patients aged above 30 and below 50 years with type 1 diabetes. SBP, systolic blood pressure; DBP, diastolic blood pressure; LOS, length of stay. *Statistical significance.

**Figure 7:**
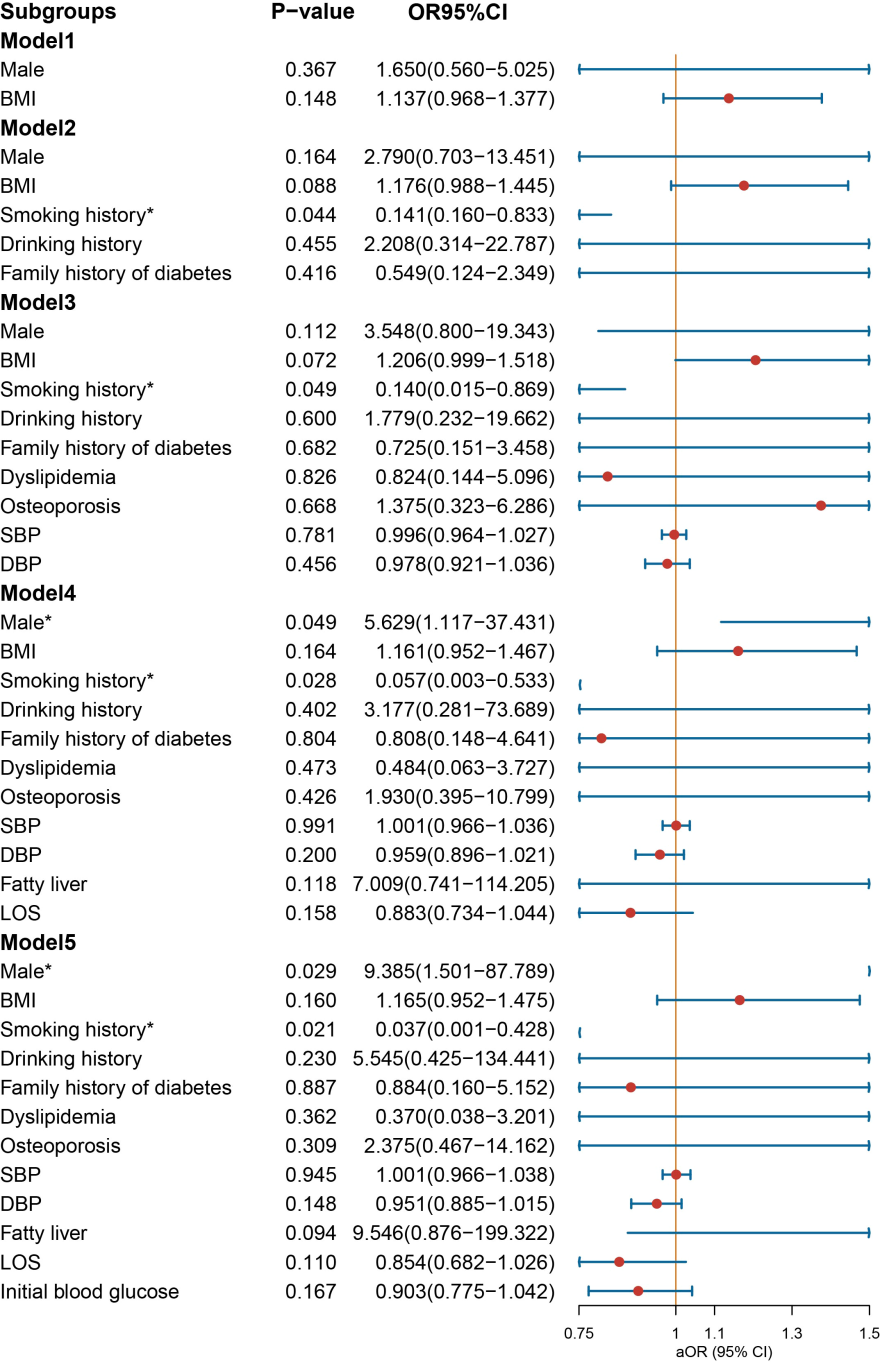
Odds ratio [95% confidence interval] of being discharged with insulin and non-insulin antidiabetic drug among patients aged above 50 with type 1 diabetes. SBP, systolic blood pressure; DBP, diastolic blood pressure; LOS, length of stay. *Statistical significance.

In the context of age-stratified multivariate regression models, the variables of BMI [OR = 1.146 (1.020–1.301), *p* = 0.028] and SBP [OR = 1.035 (1.005–1.067), *p* = 0.022] demonstrated a significant association with the prescription of NIAD in individuals below the age of 30 years (**Figure 5**). However, DBP was a protective factor for NIAD prescription [OR = 0.943 (0.906–0.980), *p* = 0.004]. After adjusting for smoking history, drinking history and family history of diabetes in Model 2, the impact of gender was no longer statistically significant. In individuals aged above 30 and below 50 years (**Figure 6**), the prescription of NIAD was found to be associated with only LOS [OR = 1.097 (1.014–1.196), *p* = 0.026] and initial blood glucose [OR = 1.078 (1.007–1.168), *p* = 0.047]. Among individuals aged above 50 years (**Figure 7**), being male [OR = 9.385 (1.501–87.789), *p* = 0.029] was identified as a risk factor for the NIAD prescription after adjusting for fatty liver and LOS factors, whereas smoking history [OR = 0.037 (0.001–0.428), *p* = 0.021] was found to be a protective factor.

## 4 Discussion

In this study, we investigated the indicators and characteristics impacting clinicians’ choice of non-insulin hypoglycemic medications for individuals diagnosed with T1D. Differences exist in the primary influencing indicators and characteristics among various populations upon admission, which doctors should prioritize when selecting treatment options. To our knowledge, there is a lack of research on the influencing factors of discharge prescription for T1D.

In our study, we found that the higher the BMI, the more likely it was to receive additional non-insulin treatment at discharge. Similar results were observed in both male and individuals younger than 30 years. Chronic pro-inflammation induced by obesity contributes to the development of insulin resistance (7), which has a negative impact on our subsequent treatment. Intensive insulin therapy has been found to correlate with increased body weight (22), hindering effective weight management in patients. Insulin dose is largely determined by body weight. If patients are overweight and exhibit inadequate compliance, the administered insulin dosage may be insufficient, thereby increasing the likelihood of hyperglycemia. In order to minimize the occurrence of these events, clinicians are more likely to add non-insulin drugs.

We found an inverse relationship between DBP and the proportion of individuals choosing non-insulin drugs combined with insulin regimens. Significant results were also observed in women and individuals younger than 30 years. However, the average amplitude of glycemic excursions exhibited a significant independent correlation with the alteration in aortic diastolic blood pressure (23). Elevated DBP is a risk factor for retinopathy (24). Patients with diabetic complications were also more commonly observed to have higher DBP (25). Patients with diabetes complications appear to have greater blood glucose variability (26, 27), thus necessitating increased focus on the management of both blood glucose and blood pressure. However, the results of this study suggest that clinicians seem to have insufficient cognition, and larger studies are needed to support our hypothesis. Several studies have indicated that certain non-insulin drugs such as metformin (28), GLP-1 agonists (29, 30), and DPP-4 inhibitors (31) do not improve blood glucose variability and diastolic blood pressure level. Hence, physicians may show limited interest in non-insulin medications for individuals with elevated DBP, as these medications may not provide optimal supplementary control over both blood glucose and blood pressure.

We observed an intriguing trend among individuals younger than 30 years: DBP emerged as a protective factor for receiving a prescription for non-insulin medications, while SBP was identified as a risk factor. This finding is often difficult to explain. Thus, we reconstructed a model to investigate the impact of pulse pressure (PP) and other factors on the likelihood of receiving prescriptions for non-insulin drugs (**Supplementary Figure 1**). After adjusting for various factors, PP consistently remained stable across the several models and emerged as a significant risk factor influencing the outcome. PP serves as an estimate of arterial stiffness (32, 33) and is considered a risk factor for cardiovascular complications among patients with T1D (33, 34). Nevertheless, certain non-insulin medications, such as metformin, exhibit the ability to impede the progression of arterial thickening and exert a certain impact on the development of atherosclerosis (35, 36). Therefore, for patients with high pulse pressure, doctors are more likely to prescribe non-insulin drugs.

Insufficient glycemic control is associated with dyslipidemia (37); thus, additional non-insulin hypoglycemic drugs are necessary to improve blood glucose management. The administration of insulin therapy continues to present the drawback of unstable regulation of blood glucose levels (22, 31, 38). Simultaneously, obesity is linked to dyslipidemia (39), while the administration of insulin therapy may induce weight gain, thereby negatively impacting the regulation of blood lipids in patients. Nevertheless, many non-insulin hypoglycemic agents, including metformin (40), GLP1 agonists (41), and SGLT-2 inhibitors (42), have been shown to have favorable effects on lipid profiles. They may be the reason why T1D patients with dyslipidemia are more likely to obtain non-insulin drug prescriptions.

The length of hospital stay is usually related to the severity of the disease. Higher blood glucose levels at the initial diagnosis often correlate with weaker blood glucose control and necessitate larger insulin doses. Hence, it may be necessary for patients to use non-insulin medications in order to decrease their insulin dosage and ensure the efficacy of blood glucose regulation.

Several studies have shown that female patients exhibit higher insulin sensitivity compared to male patients (43–45). Although insulin sensitivity decreases with age in both genders, women tend to maintain comparatively higher insulin sensitivity than men (43). To some extent, this can explain why doctors are more likely to prescribe insulin for female patients. Smoking has been found to elevate the likelihood of cardiovascular disease among individuals with T1D due to its impact on blood glucose, blood lipids, and the facilitation of endothelial dysfunction (46). Non-insulin medications exhibit certain cardiovascular advantages (4, 28, 47), thereby proving beneficial for individuals who smoke.

An ideal adjuvant treatment for T1D would improve HbA1c levels without hypoglycemia, facilitate weight loss in obese patients, and decrease the risk of diabetes-related complications (48). Treatment with insulin alone usually does not achieve this effect (4). Thus, it is not uncommon that non-insulin drugs for T2D are also used as adjunctive treatments for T1D in clinical practice (49–51). For this reason, clinicians should pay more attention to the significant indicators or factors highlighted in this article for their future work with T1D patients.

There are some limitations in our research. Firstly, it is imperative to acknowledge that the research data exclusively originate from a single-center data and potentially susceptible to inherent bias. Secondly, other potential variables that could influence the patient’s discharge treatment prescription, including the patient’s potential reluctance to utilize non-insulin medications, need to be considered. Thirdly, because of the nature of retrospective studies, we are unable to determine the specific reasons why clinical doctors prescribe or do not prescribe non-insulin drugs. Fourthly, we did not conduct a detailed analysis of individual non-insulin medications, and there are potential differences between the factors of different non-insulin drugs.

In conclusion, we identified the indicators and characteristics present at admission that exerted influence on the acquisition of non-insulin prescriptions upon discharge, alongside conducting subgroup analyses based on gender and age. During the course of treatment, the clinician should prioritize these indicators or factors in order to enhance the treatment outcome for the patient.

## Data Availability

The raw data supporting the conclusions of this article will be made available by the authors, without undue reservation.

## Supplementary material

Supplementary Figure 1 Odds ratio [95% confidence interval] of being discharged with insulin and non-insulin antidiabetic drug among patients below the age of 30 with type 1 diabetes after rebuilding the model. LOS, length of stay. *Statistical significance.
