# Supplementary figures and images for: Exploring the influencing factors of non-insulin drug prescriptions in discharged patients with type 1 diabetes
Source: Front Endocrinol (Lausanne). 2024 Sep 25;15:1381248. doi: 10.3389/fendo.2024.1381248 (PMC11467696; doi:10.3389/fendo.2024.1381248)

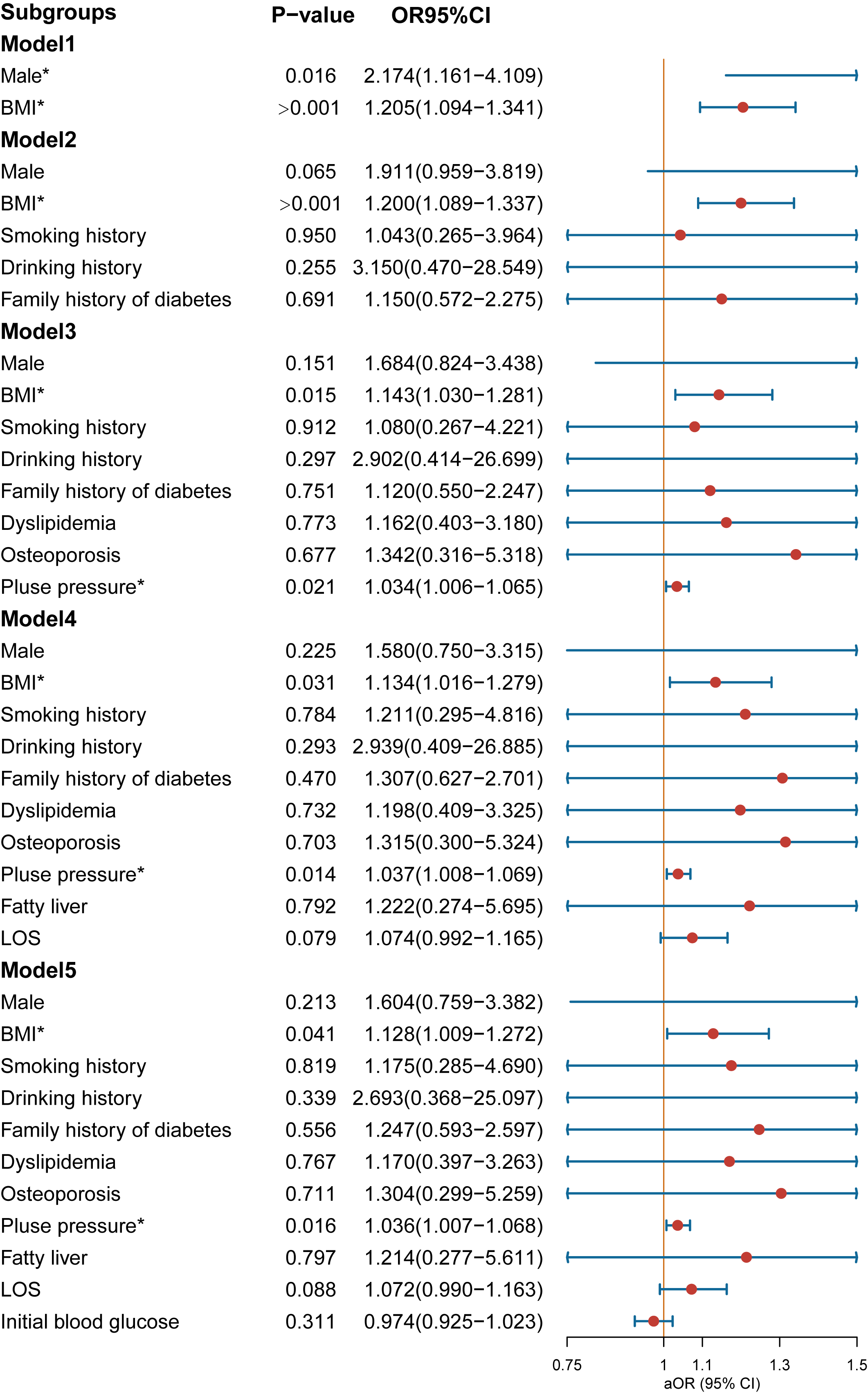

Supplement: Supplementary Figure 1 — Odds ratio [95% confidence interval] of being discharged with insulin and non-insulin antidiabetic drug among patients below the age of 30 with type 1 diabetes after rebuilding the model. LOS, length of stay. *Statistical significance. [file Image1.tif]
